# Comment on Article by Akintilo Et al.: When to Change Needles During Neuromodulator Injections—An Electron‐Microscopy Investigation Into Needle Tip Deformation

**DOI:** 10.1111/jocd.70268

**Published:** 2025-06-09

**Authors:** Yuzhi Qiu, Minghao Zhao, Jianjian Lu

**Affiliations:** ^1^ The Department of Craniomaxillofacial Surgery, Plastic Surgery Hospital Chinese Academy of Medical Sciences and Peking Union Medical College Beijing People's Republic of China

**Keywords:** cosmetic surgery, intradermal injection, plastic surgery

Dear Editor,

It was with great interest that we read the paper by Akintilo et al. [1] entitled “When to change needles during neuromodulator injections—An electron‐microscopy investigation into needle tip deformation” published on *Journal of Cosmetic Dermatology*. The article on needle deformation during neuromodulator injections highlights that repeated use leads to increased damage, recommending needle changes after three to five injections. It also challenges the notion that larger needles are more durable, as the 32G needle showed the least deformation, followed by the 31G and 30G. This offers new paths for studying factors influencing injection procedures, providing guidance for clinical practice. However, there are some issues that we would like to communicate with the authors.

First, the authors suggest changing needles after three to five uses based on the premise that new, less worn needles require less force during injections, potentially leading to greater precision. They hypothesize that as needles become dull and worn, injectors may exert more force, which could affect injection depth and hinder accuracy. We appreciate the authors' innovative insights into how needle deformation impacts the injector's force and precision. However, the article does not quantify the extent of force variations caused by needle wear. This measurement may be deemed insignificant, as inherent procedural variabilities—such as subtle fluctuations in needle insertion speed and angle—render injection force naturally dynamic in clinical practice, even when performed by the same operator [2]. Notably, a porcine cadaver study investigating needle wear revealed that while electron microscopy detected tip deformation after 12 skin penetrations, statistically significant force changes (median difference: 0.34N, *p* < 0.05) emerged only after 36 penetrations [3]. Few clinical scenarios necessitate ≥ 36 repeated injections per needle yet. Furthermore, although the authors cite Davidovic et al. [4] to link injection depth with therapeutic outcomes, this reference does not examine force‐related confounders. Thus, the assertion that needle wear‐induced force differences meaningfully compromise precision remains questionable and lacks objective evidence. Conversely, if the force differences caused by needle wear do affect the injector's tactile feedback, the timing for changing needles might be better determined by the injector's personal preference and comfort. Additionally, injection depth precision could also be achieved by selecting needles of different lengths.

Second, this article uses electron microscopy to observe the degree of needle wear to infer whether the needle remains sharp after repeated use in clinical practice. We believe that a meaningful clinical observation regarding needle sharpness is the variation in pain experienced by the same patient during needle insertions. This variation can be objectively quantified using standardized pain assessment tools, such as the Faces Pain Scale (FPS), Visual Analog Scale (VAS), and Numerical Rating Scale (NRS). If there is a correlation between the deformation of the needle and the patient's objective pain scores during insertion, then changing the needle becomes even more clinically significant.

In conclusion, we would like to voice our cordial thanks to Akintilo et al. [1] as a result of their diligent work. We commend the authors for investigating the relationship between repeated needle use and needle deformation. However, to strengthen the clinical significance of their findings, it is essential to enhance the research process. To draw more persuasive conclusions, additional studies with larger sample sizes and clinically relevant variables are necessary.

## Author Contributions

Yuzhi Qiu is responsible for manuscript writing. Minghao Zhao and Jianjian Lu are responsible for topic selection, theoretical guidance, and coordination.

## Ethics Statement

The authors have nothing to report.

## Conflicts of Interest

The authors declare no conflicts of interest.

## Data Availability

The authors have nothing to report.
